# Robustness asymmetry under edge failures: A unified framework based on structural controllability and power domination

**DOI:** 10.1371/journal.pone.0361399

**Published:** 2026-10-28

**Authors:** Bader Alwasel

**Affiliations:** Unit of Scientific Research, Applied College, Qassim University, Buraydah, Saudi Arabia; Dr Shakuntala Misra National Rehabilitation University, INDIA

## Abstract

Modern complex networks require effective mechanisms for network control and monitoring to ensure reliable operation under failures. Structural controllability and power domination provide fundamental frameworks for assessing these capabilities, yet a systematic comparison of their robustness under random failures remains limited. Unlike prior studies that typically examine structural controllability and power-domination-based monitoring separately, this work presents a unified framework for analyzing their robustness under identical edge-failure conditions in Erdős–Rényi random graphs. Using a simulation-based evaluation across a broad parameter space of network sizes, edge densities, and edge-survival probabilities, together with Monte Carlo estimation of the expected monitoring coverage and driver-node requirements under random edge failures, the analysis evaluates degradation through sensitivity, balance, and gap metrics. The results reveal a configuration-dependent robustness asymmetry, with structural controllability exhibiting its most pronounced relative vulnerability under sparse and severely degraded network conditions. Notably, following edge failures, the two frameworks are reevaluated through different structural mechanisms: the power dominating set is recomputed on the degraded network to determine the updated monitoring-node requirement, whereas the maximum matching is recomputed to determine the updated driver-node requirement for structural controllability. Network density also plays a critical role, with sparse networks exhibiting higher vulnerability and dense networks showing greater resilience. These findings indicate that, for the considered Erdős–Rényi random-network model under independent random edge failures, the stronger relative vulnerability of structural controllability is concentrated primarily in sparse and severely degraded network configurations.

## 1 Introduction

Networked systems form the backbone of many critical infrastructures, including power grids, communication networks, and transportation systems. Ensuring their reliable operation requires both the ability to influence system dynamics through control and to infer system states through monitoring. In practice, these systems may be exposed to structural disruptions, such as random link failures that modify network connectivity. Such disruptions can significantly affect both structural controllability and monitoring processes, making it essential to understand how these capabilities deteriorate under failure conditions. Understanding the comparative robustness of structural controllability and power-domination-based monitoring is particularly important in modern networked infrastructures, including cyber-physical systems, smart grids, transportation systems, and distributed communication networks, where structural disruptions may simultaneously affect both control and monitoring capabilities. In real-world environments, failures often occur unpredictably, for example through random link removals or component malfunctions. Among various failure models, random edge removal provides a realistic abstraction of many practical scenarios. The robustness of complex networks under such failures has been widely studied, showing that structural disruptions can significantly impact connectivity and functionality [1–3]. This raises a fundamental question: how do structural changes induced by failures influence the ability to control and monitor a network? More specifically, it is important to determine whether structural controllability and power-domination-based monitoring degrade in a similar manner, or whether one is inherently more sensitive to structural perturbations.

Structural controllability provides a graph-theoretic framework for determining whether a system can be driven to desired states using external inputs [4,5]. In this framework, the minimum number of driver nodes is obtained via maximum matching, where unmatched nodes must be directly controlled. Formally, if |*M*| denotes the size of a maximum matching in the directed representation of the network, the number of driver nodes $N_{D}$ is given by


$$\begin{array}{c} N_{D}=\max\{1,n-|M|\}, \end{array}$$

Subsequent work has established broader control principles in complex networks and highlighted the role of network structure in determining controllability [6–8]. Recent studies have also examined controllability in cyber-physical and evolving networks, emphasizing the role of critical nodes and edges under structural disruptions [9,10].

Complementary to control, power domination provides a propagation-based monitoring framework in which monitoring units are placed and propagation rules iteratively extend network coverage [11]. This framework captures how a subset of nodes can monitor the entire system, making it particularly relevant for monitoring applications such as power grids. The power domination number represents the minimum number of monitoring units required to achieve complete network monitoring.

Although both frameworks address fundamental aspects of network functionality, they rely on fundamentally distinct mechanisms: matching-based control and propagation-based monitoring. In practical networked systems, these capabilities are inherently coupled, as effective operation requires both control inputs and monitoring. Consequently, a systematic comparison of the robustness of structural controllability and power-domination-based monitoring under structural disruptions is essential for characterizing their relative vulnerability and guiding the design of resilient networked systems.

In this study, robustness refers to the ability of structural controllability and power-domination-based monitoring to maintain their performance under edge failures. Robustness is assessed through complementary measures of degradation and sensitivity, where degradation quantifies the loss of performance after edge failures and sensitivity characterizes the extent of performance degradation relative to the level of structural damage.

Structural observability is often treated as the dual counterpart of structural controllability. However, the present work focuses on power domination, which provides a propagation-based monitoring framework rather than a general formulation of structural observability. Accordingly, the comparison in this study is between structural controllability and power-domination-based monitoring. This distinction enables a more meaningful comparison between fundamentally different control and monitoring paradigms under structural degradation.

While both structural controllability and power-domination-based monitoring have been extensively studied, they have largely been examined in isolation, with comparatively limited and fragmented efforts toward systematically comparing their robustness within a unified failure framework. Prior studies on structural controllability and structural observability have explored their relationship and highlighted their distinct structural and functional roles in complex networks [12,13]. Existing research has shown that structural controllability is sensitive to structural perturbations such as node and edge failures, where disruptions can increase the number of required driver nodes and reduce control efficiency [14,15]. Similarly, power-domination-based monitoring depends strongly on network connectivity, where structural changes can limit propagation and reduce monitoring effectiveness. Prior studies have also quantified structural controllability robustness across different network structures [16]. However, recent work has highlighted limitations of purely structural models, showing that neglecting system dynamics may lead to overestimation of control and observation requirements [17].

Taken together, these findings suggest that structural controllability and power-domination-based monitoring may exhibit distinct robustness behaviors under structural disruptions. Nevertheless, a systematic and quantitative comparison of structural controllability and power-domination-based monitoring under identical random edge failure conditions remains limited. In particular, the comparative response of these two frameworks in terms of degradation behavior, scalability, and sensitivity has received comparatively limited attention. The principal contribution of this work is the development of a unified comparative framework for systematically comparing structural controllability and power-domination-based monitoring under identical random edge-failure conditions. To address this gap, this paper seeks to answer the following research questions:

- How do structural controllability and power-domination-based monitoring behave under random edge failures?
- Which framework exhibits greater sensitivity to such failures?
- How do control and monitoring metrics scale with network size under degraded conditions?
- How does network density influence degradation behavior across different failure levels?

The main contributions of this work are summarized as follows:

- A unified framework for systematically comparing structural controllability and power-domination-based monitoring under identical random failure conditions.
- A set of robustness metrics capturing sensitivity, degradation, and control–monitoring balance.
- A large-scale empirical evaluation revealing distinct robustness patterns across varying network sizes and densities.
- Identification and quantitative characterization of a robustness imbalance between structural controllability and power-domination-based monitoring under structural disruptions.

The remainder of this paper is organized as follows. The Related Work section reviews related work on structural controllability, power domination, and network robustness under failures. The Preliminaries and Definitions section introduces the preliminaries and fundamental definitions. The Structural Controllability and Power-Domination-Based Monitoring Evaluation section presents the evaluation framework for analyzing structural controllability and power-domination-based monitoring under random edge failures. The Results and Analysis section reports the experimental results and analysis. The Discussion section discusses the implications of the findings and provides further insights. Finally, the Conclusion section concludes the paper and outlines directions for future work.

## 2 Related work

Structural controllability has been extensively studied in complex networks. The foundational work of Lin [4] established the structural conditions for controllability, while Liu *et al.* [5] extended this framework to complex networks using maximum matching. In this formulation, the minimum number of driver nodes is determined by the size of the maximum matching, where unmatched nodes must be directly controlled. Subsequent studies demonstrated that network structure determines controllability properties, including exact controllability and the influence of nodal dynamics [6–8]. In particular, Menichetti *et al.* [18] showed that the density of low in-degree and out-degree nodes plays a key role in determining the number of driver nodes, highlighting the structural dependence of controllability. This result provides a structural explanation for degradation phenomena, as edge failures tend to reduce node degrees and increase the fraction of low-degree nodes in the network. More recent work has further explored controllability from structural and dynamical perspectives, including multilayer and temporal networks [6,16]. In addition to foundational studies, several works have explored the relationship between controllability and observability in complex networks. Liu *et al.* [12] extended structural analysis to observability, while Yan *et al.* [13] introduced a unified perspective linking control and observation through a spectrum of network behaviors. Furthermore, Ruths and Ruths [19] highlighted the diversity of control profiles across networks, emphasizing that structural properties alone do not fully characterize controllability. The effect of structural perturbations on controllability has also been widely investigated. Node and edge removals reduce matching size and increase the number of required driver nodes, indicating that controllability is sensitive to failures and targeted attacks [14,15,20,21]. These effects are particularly significant in sparse and heterogeneous networks, where critical nodes and edges dominate system behavior. Notably, Mengiste *et al.* [22] provided a detailed analysis of the impact of edge pruning on structural controllability, demonstrating that link removal can significantly degrade controllability depending on the pruning strategy and network topology. Their findings provide important insight into failure-induced degradation; however, their analysis does not consider domination-based monitoring frameworks such as power domination. In a related direction, Xiao *et al.* [23] investigated the robustness of network controllability under malicious attacks and proposed strategies to enhance resilience, highlighting the impact of targeted disruptions on control performance. Recent studies have further extended robustness analysis to hybrid failure scenarios involving simultaneous node and edge disruptions [24]. Such approaches introduce hybrid connectivity measures to characterize the combined impact of failures and propose protection strategies for improving network resilience. While these models broaden the scope of robustness analysis, their implications for controllability and monitoring frameworks remain relatively underexplored. Robustness analysis has also been extended to cyber-physical systems, where networked control and monitoring processes are jointly affected under diverse failure scenarios [9,10,25]. Moreover, comparative analyses have quantified controllability robustness across different network structures [16]. While maximum-matching-based approaches have proven effective for structural controllability analysis, recent studies have highlighted certain limitations when system dynamics are not explicitly considered. In particular, Leitold *et al.* [17] showed that neglecting intrinsic node dynamics, such as self-loops and interaction effects, may lead to overestimation of control and observation requirements. These findings emphasize the importance of extending purely structural representations toward more comprehensive state-space models. Nevertheless, structural approaches remain widely adopted in large-scale network analysis due to their scalability, analytical tractability, and suitability for studying topology-driven robustness properties.

From a propagation-based monitoring perspective, power domination has been widely used to model monitoring processes in networks, particularly in power systems [11,26,27]. This framework captures how a subset of nodes can observe the entire network through propagation rules and has direct applications in modern monitoring systems such as phasor measurement units (PMUs) [28]. Recent work has focused on computational aspects and efficient placement strategies for large-scale networks, as well as variants of domination-based monitoring under structural constraints [29]. Furthermore, observability analysis has been extended to cyber-physical systems, particularly in electric power grids, where monitoring depends on both the physical infrastructure and the underlying communication network [30]. These developments highlight the practical relevance of graph-based monitoring models, such as power domination, while also indicating that real-world monitoring is influenced by interdependent network structures. Structural disruptions can significantly affect propagation-based monitoring by blocking propagation paths and increasing the size of the required monitoring set. In networks with articulation points or limited connectivity, these effects become more pronounced. Studies have also explored the relationship between structural controllability and power domination in random networks, highlighting potential connections between control and monitoring processes [31]. Despite these advances, existing studies differ in scope from the present work. Previous research has primarily focused on either structural controllability or power domination independently, or on their conceptual relationship, without providing a unified quantitative comparison under identical random edge-failure realizations. Moreover, robustness has typically been evaluated using framework-specific measures, making direct comparisons difficult. In contrast, the present study adopts a unified comparative framework together with complementary robustness metrics to enable a systematic quantitative comparison of structural controllability and power-domination-based monitoring under identical random edge-failure conditions.

Taken together, these studies highlight significant progress in understanding network control and monitoring. Nevertheless, a systematic understanding of the comparative robustness of structural controllability and power-domination-based monitoring under identical random edge-failure conditions remains limited.

## 3 Preliminaries and definitions

### 3.1 Graph model and notation

Let *G* = (*V*, *E*) denote an undirected graph, where *V* is the set of nodes with |*V*| = *n*, and *E* is the set of edges. The structure of the network is represented by its adjacency matrix $A\in \{0,1{\}}^{n\times n}$, where $A_{ij}=1$ if there exists an edge between nodes *i* and *j*, and $A_{ij}=0$ otherwise. Throughout this paper, we consider simple, unweighted graphs without self-loops or parallel edges.

The network topology is generated using the Erdős–Rényi (ER) random graph model [32], denoted as *G*(*n*,*p*), where *n* is the number of nodes and *p* is the edge formation probability. To ensure a consistent baseline for the comparative robustness analysis, only connected graph realizations are considered as the initial networks. Specifically, graph instances are repeatedly generated until a connected realization is obtained, with a maximum of 5000 trials per configuration to ensure a high probability of obtaining such realizations, particularly for low edge densities. To evaluate robustness under structural degradation, random edge failures are introduced, where each edge is independently retained with probability q∈[0,1], and *q* denotes the edge survival probability. The resulting perturbed graph is denoted by $G_{q}=(V,E_{q})$, where $E_{q}\subseteq E$. Following random edge failures, the degraded graph may become disconnected. In the proposed framework, both structural controllability and power domination are evaluated directly on the resulting degraded graph, regardless of the number of connected components. Accordingly, the reported metrics quantify the structural impact of edge failures on structural controllability and power-domination-based monitoring rather than the operational status or failure criterion of a specific physical system.

For structural controllability analysis, the undirected graph is transformed into a directed representation by assigning a direction to each edge, as required by the maximum matching framework used to identify driver nodes [5]. For the representative robustness evaluation, each edge is assigned a direction uniformly at random, and this orientation is preserved after edge failures by retaining the directions of the surviving edges. In the separate Monte Carlo estimation of expected post-failure quantities, an independent random orientation is assigned to the surviving edges of each failure realization.

Power domination is evaluated directly on the undirected graph using propagation rules. The observed set *O*(*S*) is computed iteratively: initially selected nodes (the PDS) observe themselves and their neighbors; then, any observed node with exactly one unobserved neighbor propagates observation to that neighbor; this process continues until no further nodes can be observed.

The experimental framework evaluates robustness across a comprehensive range of network sizes, edge densities, and edge-survival probabilities. Across the considered parameter configurations, the adopted structural controllability and power domination metrics are evaluated to provide a systematic characterization of degradation patterns. For each parameter configuration (*n*,*p*,*q*), one connected Erdős–Rényi graph is generated. For the specified edge-survival probability *q*, Monte Carlo simulations with *MC* = 200 independent edge-failure realizations are performed on this base graph. In each Monte Carlo trial, the same failed undirected network is used for both the power-domination-based monitoring and structural controllability evaluations. For the structural controllability calculation within the Monte Carlo estimation, an independent random orientation is assigned to the surviving edges of each failed realization. The Monte Carlo simulations are used to estimate the expected monitoring coverage of the original power dominating set and the expected post-failure driver-node ratio. Separately, the degradation metrics, sensitivity coefficients, and comparative robustness measures are evaluated using a representative failure realization in which one random orientation is assigned to the original graph and preserved for all surviving edges after failure, as summarized in Algorithm 3.

### 3.2 Structural controllability and power domination

Structural controllability provides a graph-theoretic framework for determining whether a dynamical system can be fully controlled through external inputs based on its underlying structure. Consider a linear time-invariant system described by


$$\begin{array}{c} \dot{x}(t)=Ax(t)+Bu(t), \end{array}$$

where *A* represents the system matrix and *B* denotes the input matrix. According to the framework introduced by Lin [4], controllability depends only on the zero–nonzero structure of *A* and *B*. In complex networks, this concept can be analyzed using a directed graph representation, where nodes correspond to state variables and edges represent interactions. Liu *et al.* [5] showed that the minimum number of driver nodes required to control a network can be determined by mapping the problem to a maximum matching in the directed graph. A matching is defined as a set of directed edges such that no two edges share a common starting or ending node. Let |*M*| denote the size of a maximum matching. The minimum number of driver nodes $N_{D}$ is then given by


$$\begin{array}{c} N_{D}=\max\{1,n-|M|\}, \end{array}$$

Unmatched nodes in the maximum matching correspond to driver nodes that must be directly controlled to ensure full network controllability.

In contrast, power domination is a graph-theoretic framework used to model propagation-based monitoring in networks, particularly in electrical power systems [11]. The objective is to determine a minimum set of nodes, called the power dominating set (PDS), from which the entire network can be observed through a set of propagation rules. Given a graph *G* = (*V*,*E*) and an initial set of monitored nodes S⊆V, the observation process proceeds iteratively in two stages. First, in the domination step, all nodes in *S* and their neighbors become observed. Then, in the propagation step, if an observed node has exactly one unobserved neighbor, that neighbor becomes observed. This process continues until no further nodes can be observed. The goal is to identify a minimum PDS such that all nodes in the network are eventually observed, and the size of this set is referred to as the power domination number. The power domination number, denoted by $\gamma_{P}(G)$, is defined as the cardinality of a minimum power dominating set. Since computing $\gamma_{P}(G)$ exactly is NP-hard, the large-scale experiments reported in this paper employ a greedy selection procedure followed by a pruning step to obtain an approximate PDS size. The accuracy of this approximation is validated separately using exhaustive exact computation on smaller networks, as described in the Validation of the Greedy-Pruned Power Dominating Set Algorithm subsection.

These two frameworks rely on fundamentally different mechanisms, namely matching-based control and propagation-based monitoring, which lead to distinct structural responses under network disruptions.

## 4 Structural controllability and power-domination-based monitoring evaluation

This section presents the evaluation framework used to quantify the robustness of structural controllability and power-domination-based monitoring under random edge failures, based on the structural controllability and power domination models introduced earlier. The network topology is generated using the Erdős–Rényi (ER) random graph model. The experiments are conducted on networks with sizes n∈{50,100,200,500,1000} and edge probabilities p∈{0.1,0.3,0.5,0.7}, covering a range of network densities from sparse to relatively dense structures. This enables a systematic evaluation of how the robustness of structural controllability and power-domination-based monitoring varies across different levels of network connectivity. To evaluate robustness under structural degradation, random edge failures are introduced. Each edge is independently retained with probability q∈{0.1,0.3,0.5,0.7,0.9,1}, representing different levels of failure severity, ranging from highly degraded networks to fully intact structures. For clarity and reproducibility, the main computational procedures are summarized in Algorithms 1–3.

For each connected network realization, structural controllability is evaluated by converting the undirected network into a directed representation, where each edge is assigned a random direction. For the representative-realization robustness metrics, one random orientation is generated for the original network and preserved after edge failures by retaining the directions of the surviving edges. Accordingly, the resulting changes in controllability are solely attributed to edge removals rather than repeated random reorientation. Separately, Monte Carlo simulations (*MC* = 200) are performed to estimate the expected post-failure driver-node ratio, where an independent random orientation is assigned to each failed realization, consistent with the implemented simulation procedure. The remaining robustness metrics are then computed using this representative failure realization. Maximum matching (via Hopcroft–Karp) is then applied to identify the controllability structure. The number of unmatched nodes determines the minimum set of driver nodes required to control the network. The procedure is summarized in Algorithm 1.

**Algorithm 1 Driver Node Set via Maximum Matching**

**Require:** Directed adjacency matrix $A_{dir}$ of graph *G*(*V*,*E*)

**Ensure:** Set of driver nodes 𝒟⊆V

1: Construct bipartite graph $\left(U,W,E^{\prime }\right)$ from $A_{dir}$, where *U* and *W* denote left and right copies of *V*

2: Compute maximum matching ℳ using the Hopcroft–Karp algorithm

3: Identify unmatched nodes in *W*

4: 𝒟← set of unmatched nodes in *W*

5: **if** 𝒟=∅ **then**

6:  Select an arbitrary node and add it to 𝒟

7: **end if**

8: **return** 𝒟

Accordingly, structural controllability is quantified using the driver-node ratio


$$\begin{array}{c} r_{D}=\frac{N_{D}}{n}, \end{array}$$

where $N_{D}$ is the number of driver nodes. In addition, the matched-node ratio is defined as


$$\begin{array}{c} r_{M}=\frac{N_{M}}{n}, \end{array}$$

where $N_{M}=n-N_{D}$ denotes the number of matched nodes.

Power-domination-based monitoring is evaluated using the PDS framework. A greedy algorithm is employed to iteratively select nodes that maximize network coverage, followed by a pruning step to obtain a greedy-pruned PDS. When multiple candidate nodes provide the same increase in observed nodes, ties are resolved deterministically by selecting the candidate with the highest degree. The procedure is summarized in Algorithm 2.

**Algorithm 2 Greedy Power Domination Set (PDS) with Pruning**

**Require:** Adjacency matrix *A* of graph *G*(*V*,*E*), |*V*| = *n*

**Ensure:** A greedy-pruned power dominating set S⊆V

1: S← set of isolated nodes in *G*

2: Compute the observed set *O*(*S*)

3: **while** |*O*(*S*)| < *n* **do**

4:  Select node v∈V⧵S that maximizes the increase in |*O*(*S*)|

5:   breaking ties by selecting the candidate with the highest degree

6:  S←S∪{v}

7:  Update *O*(*S*)

8: **end while**

9: **Pruning phase**

10: **for** each s∈S in reverse order of addition **do**

11:  **if** O(S⧵{s})=V **then**

12:   S←S⧵{s}

13:  **end if**

14: **end for**

15: **return** *S*

An illustrative example of the computed PDS on a representative network is shown in Fig 1, where a small subset of nodes enables monitoring of the entire network through propagation. Power-domination-based monitoring is quantified using the PDS ratio


$$\begin{array}{c} r_{P}=\frac{\left|S\right|}{n}, \end{array}$$

where *S* is the resulting power dominating set. The propagation process is then applied to determine the set of observed nodes *O*(*S*) according to the standard power domination rules. The observed fraction is defined as


$$\begin{array}{c} r_{O}=\frac{\left|O(S)\right|}{n}, \end{array}$$

which represents the proportion of the network that can be monitored from the selected PDS.

**Fig 1:**
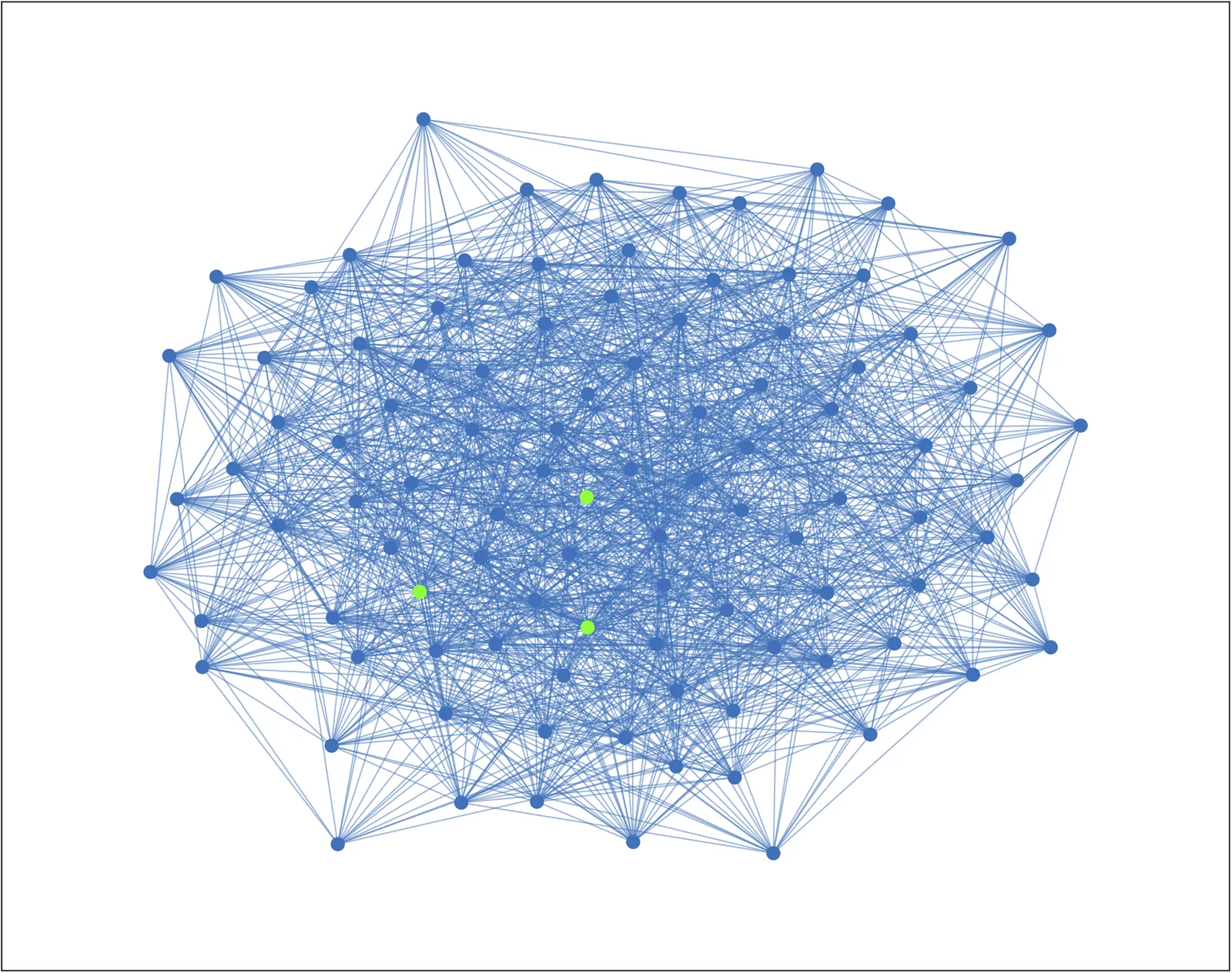
Original Erdős–Rényi network (*n* = 100, *p* = 0.3) with highlighted power domination set (PDS) nodes. The resulting PDS contains 3 monitoring nodes that enable power-domination-based monitoring across the network.

### 4.1 Validation of the greedy-pruned power dominating set algorithm

Since computing the theoretical power domination number exactly is NP-hard, the large-scale experiments reported in this paper employ a greedy selection procedure followed by a pruning step to approximate the minimum power dominating set (PDS) for large networks. To verify that the reported robustness trends are not artifacts of this approximation, we performed an independent validation study on small connected Erdős–Rényi networks, where exhaustive exact computation is computationally feasible.

The validation considered network sizes *n* = {10,12,14}, edge probabilities *p* = {0.1,0.3,0.5,0.7}, edge-survival probabilities *q* = {0.1,0.3,0.5,0.7,0.9}, and 20 independently generated graph instances for each (*n*,*p*) configuration. Each graph was evaluated under all five edge-survival probabilities, producing 3×4×20×5=1,200 before–after validation cases. In each case, the greedy-pruned PDS was compared with the exhaustive exact solution both before and after edge failures, resulting in 2×1,200=2,400 evaluated PDS solutions.

The validation results are summarized in Table 1. Across all tested instances, the greedy-pruned algorithm produced the same minimum power dominating set size as the exhaustive exact search, yielding a 100% optimality rate, zero node error, an approximation ratio of 1.0, and identical values of the PDS-size requirement change obtained from the greedy-pruned and exact solutions. These results indicate that, for all tested network sizes where exact computation is computationally feasible, the greedy-pruned algorithm reproduces the minimum power domination number. Therefore, the reported robustness trends are unlikely to be artifacts introduced by the adopted approximation algorithm and instead reflect the underlying structural properties of the evaluated networks.

**Table 1:** Validation results for the greedy-pruned PDS algorithm using exhaustive exact computation.

Metric	Value
Network sizes	10,12,14
Evaluated PDS solutions	2400
Agreement with exact PDS size	100%
Mean node error	0
Maximum node error	0
Approximation ratio	1.0
Identical PDS-size requirement changes	100%

To assess robustness, both structural controllability and power-domination-based monitoring are evaluated before and after edge failures. For power-domination-based monitoring, two scenarios are considered: first, applying the original PDS to the degraded network to evaluate the robustness of the existing monitoring configuration; second, recomputing the PDS after failures to obtain a reconfigured monitoring set for the degraded network and quantify the corresponding post-failure monitoring-resource requirements. In parallel, structural controllability is reevaluated after failures by recomputing the maximum matching on the degraded network to determine the updated driver-node requirement. This distinction reflects the inherent characteristics of the adopted structural controllability and power domination frameworks rather than an experimental preference. Accordingly, each framework is evaluated according to its standard formulation, allowing their respective robustness characteristics to be compared while highlighting the practical implications of their different post-failure evaluation mechanisms. In the adopted structural controllability framework, recomputing the maximum matching updates the minimum driver-node requirement imposed by the degraded network structure. Accordingly, the controllability analysis quantifies the resulting post-failure driver-node requirement, whereas the power-domination-based monitoring analysis additionally evaluates the original and reconfigured monitoring configurations after failure by first applying the original power domination set *S* to the failed network to determine the corresponding observed set (*O*_old_), and subsequently recomputing a new power domination set $S_{f}$ on the failed network to determine the corresponding observed set (*O*_after_). This distinction separates the immediate monitoring degradation from the monitoring configuration obtained through PDS recomputation. The impact of edge failures on power-domination-based monitoring is illustrated in Fig 2, where random edge removals reduce connectivity and limit the propagation capability of the original PDS configuration.

**Fig 2:**
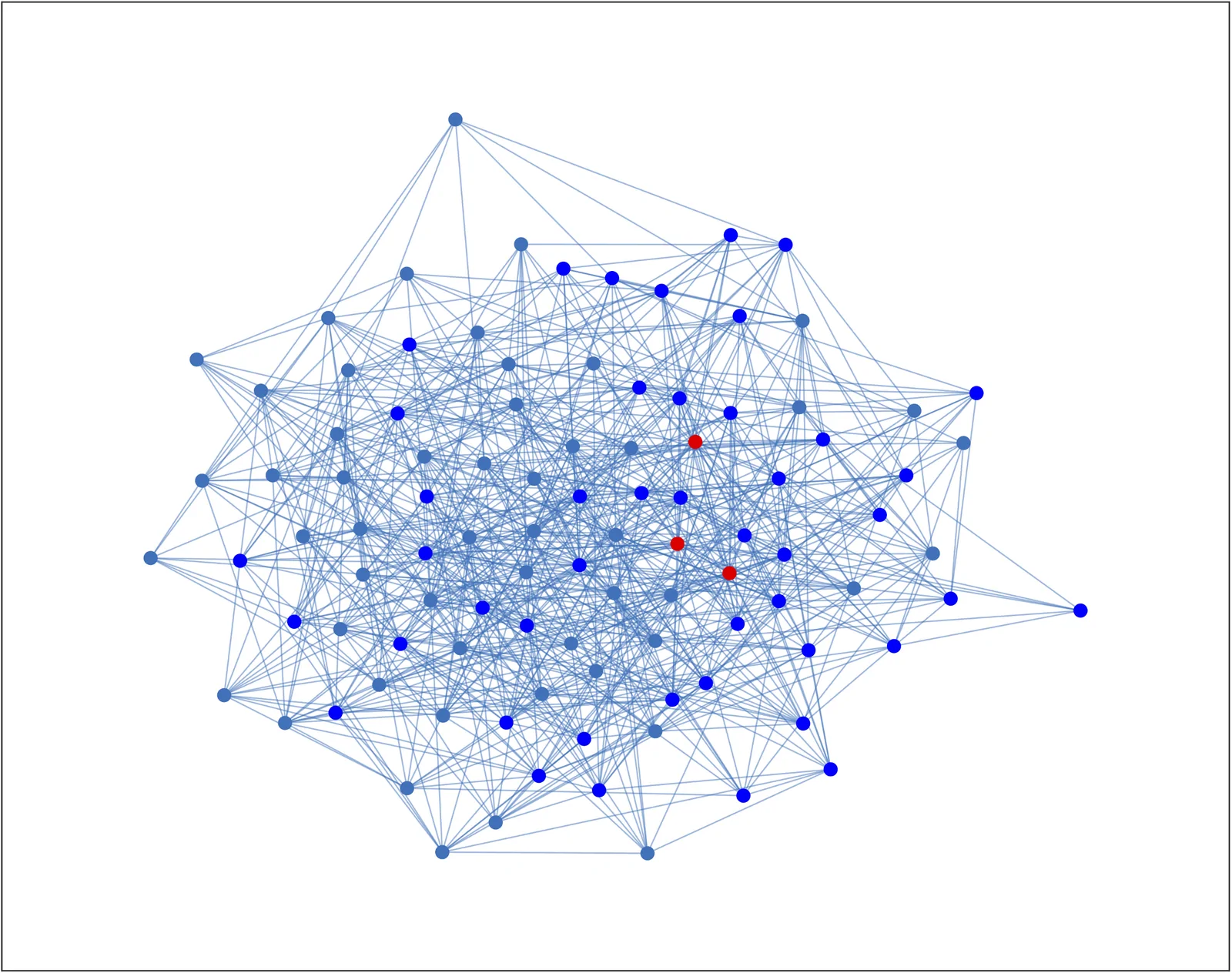
Failed-edge sample of the Erdős–Rényi network after random edge failures (*n* = 100, *p* = 0.3, *q* = 0.5). The degraded network retains 740 edges. Using the original PDS (size = 3), only 45 of 97 non-PDS nodes remain observable (shown in dark blue), illustrating the effect of connectivity degradation on power-domination-based monitoring.

The performance of the original monitoring configuration after edge failures is evaluated by applying the original power dominating set *S* to the failed network, yielding *O*_old_. This quantity directly characterizes the loss of monitoring coverage without reconfiguration. Separately, recomputing the power dominating set on the failed network yields $S_{f}$, whose size quantifies the post-failure monitoring-resource requirement. Accordingly, the non-reconfigured monitoring coverage and the monitoring-node requirement after recomputation are evaluated as distinct post-failure quantities. In the adopted structural controllability framework, post-failure controllability is evaluated by recomputing the maximum matching on the degraded network, thereby determining the updated minimum driver-node requirement imposed by the degraded network structure. This provides a consistent post-failure comparison between the recomputed driver-node requirement and the recomputed monitoring configuration, while separately reporting the performance of the original monitoring configuration. This distinction reveals a robustness imbalance between structural controllability and power-domination-based monitoring under structural disruptions and helps explain their different resilience characteristics. The overall evaluation framework is summarized in Algorithm 3.

**Algorithm 3 Robustness Evaluation under Random Edge Failures**

**Require:** Network size *n*, edge probability *p*, survival probability *q*, and Monte Carlo trials *MC*

**Ensure:** Expected non-reconfigured monitoring coverage $𝔼[|O_{old}|]$, expected driver-node ratio $𝔼[r_{D,f}]$, post-failure change metrics $\Delta_{D}$, $\Delta_{M}$, $\Delta_{P}$, $\Delta_{O}$, sensitivity coefficients $\kappa_{D}$, $\kappa_{M}$, $\kappa_{P}$, $\kappa_{O}$, and comparison metrics Γ and Λ

1: Generate a connected ER graph *G*(*V*,*E*) with edge probability *p*

2: Compute the initial greedy-pruned PDS *S* and its observed set *O*_before_

3: Store |*E*|, |*S*|, |*O*_before_|, and $\left|O_{before}|-|S\right|$

4: **Monte Carlo estimation of expected post-failure quantities**

5: Initialize $Sum_{O}\leftarrow 0$ and $Sum_{r_{D}}\leftarrow 0$

6: **for** *t* = 1 to *MC* **do**

7:  Generate $G_{f}^{\left(t\right)}$ by independently retaining each edge with probability *q*

8:  Compute $O_{old}^{\left(t\right)}$ by applying the original PDS *S* to $G_{f}^{\left(t\right)}$

9:  Assign an independent random orientation to the surviving edges of $G_{f}^{\left(t\right)}$

10:  Compute the post-failure driver-node ratio $r_{D,f}^{\left(t\right)}$

11:  $Sum_{O}\leftarrow Sum_{O}+|O_{old}^{\left(t\right)}|$

12:  $Sum_{r_{D}}\leftarrow Sum_{r_{D}}+r_{D,f}^{\left(t\right)}$

13: **end for**

14: $𝔼[|O_{old}|]\leftarrow Sum_{O}/MC$

15:  $𝔼[r_{D,f}]\leftarrow Sum_{r_{D}}/MC$

16: **Single failure-realization post-failure evaluation**

17: Generate one random orientation of the original edges of *G* and preserve it

18: Compute the initial driver-node set 𝒟 and matched-node count $N_{M}$

19: Generate one failed graph $G_{f}$ by independently retaining each edge with probability *q*

20: Apply the preserved original orientation to the surviving edges of $G_{f}$

21: Store $\left|E_{f}\right|$

22: Compute the post-failure driver-node set ${𝒟}_{f}$ and matched-node count $N_{M,f}$

23: Compute a new greedy-pruned PDS $S_{f}$ and its observed set *O*_after_ on $G_{f}$

24: Compute *O*_old_ by applying the original PDS *S* to $G_{f}$ to quantify the non-reconfigured monitoring coverage

25: $\Delta_{D}\leftarrow (|{𝒟}_{f}|-|𝒟|)/n$

26: $\Delta_{M}\leftarrow (N_{M}-N_{M,f})/\max(N_{M},1)$

27: $\Delta_{P}\leftarrow (|S_{f}|-|S|)/n$

28: $\Delta_{O}\leftarrow [(|O_{before}|-|S|)-(|O_{after}|-|S_{f}|)]/\max(|O_{before}|-|S|,1)$

29: $\phi \leftarrow (|E|-|E_{f}|)/|E|$

30: **if** ϕ>0 **then**

31:  $\kappa_{D}\leftarrow \Delta_{D}/\phi$

32:  $\kappa_{M}\leftarrow \Delta_{M}/\phi$

33:  $\kappa_{P}\leftarrow \Delta_{P}/\phi$

34:  $\kappa_{O}\leftarrow \Delta_{O}/\phi$

35: **else**

36:  Sensitivity coefficients are undefined (ϕ=0)

37: **end if**

38: $\Gamma \leftarrow |{𝒟}_{f}|/\max(|S_{f}|,1)$

39: $\Lambda \leftarrow \Delta_{D}-\Delta_{P}$

40: **return** $𝔼[|O_{old}|]$, $𝔼[r_{D,f}]$, $\Delta_{D}$, $\Delta_{M}$, $\Delta_{P}$, $\Delta_{O}$, $\kappa_{D}$, $\kappa_{M}$, $\kappa_{P}$, $\kappa_{O}$, Γ, and Λ

Fig 3 illustrates the reconfiguration of power-domination-based monitoring through recomputation of the PDS, where a new monitoring set is obtained for the degraded network.

**Fig 3:**
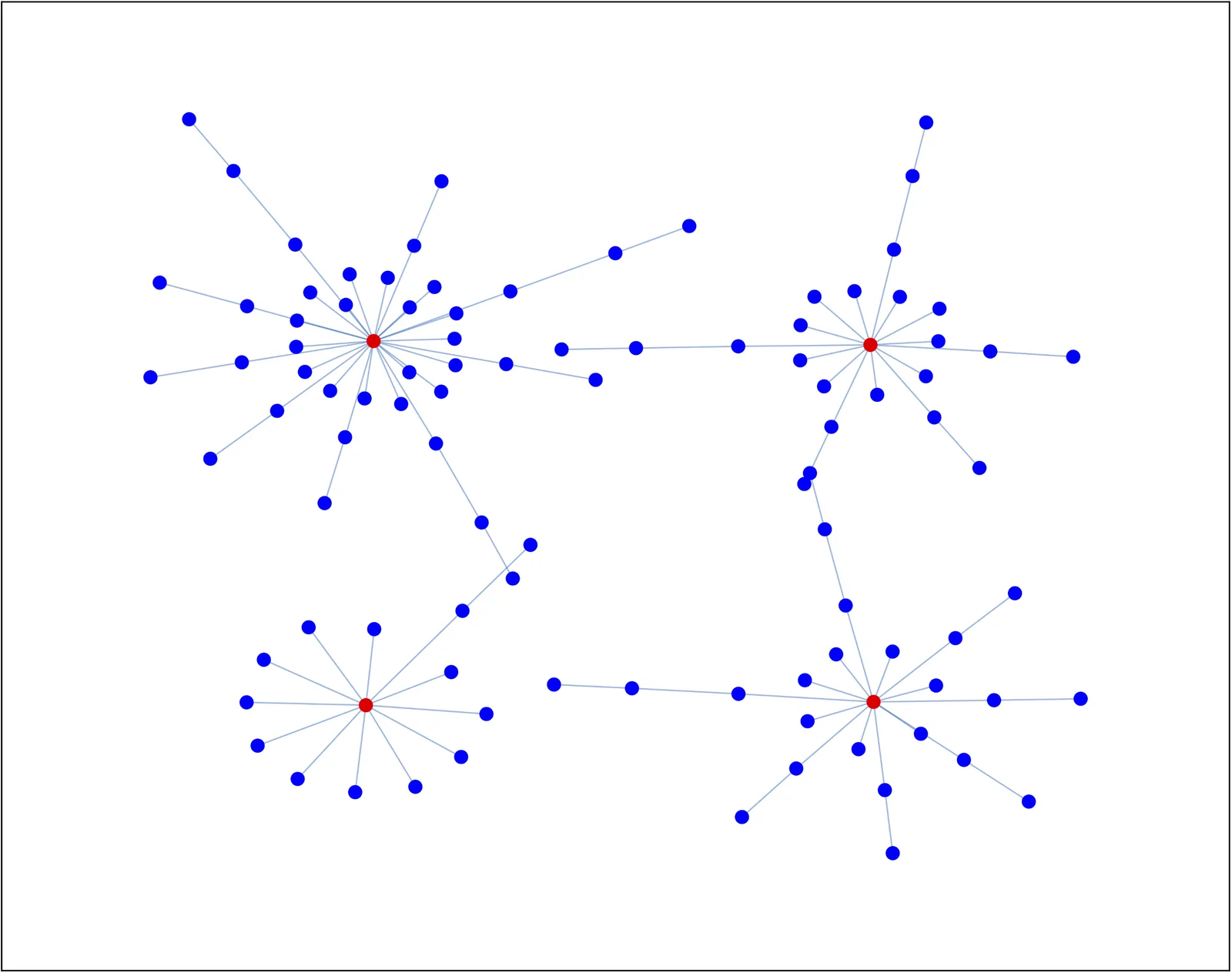
Power domination after failure using a recomputed PDS configuration. The recomputed PDS provides a monitoring configuration for the degraded network. PDS size = 4, Coverage = 96/96.

The post-failure changes are defined separately for each metric because the evaluated quantities have different interpretations. An increase in the number of required driver nodes or power dominating set (PDS) nodes represents degradation, whereas a decrease in the number of matched or observed nodes represents degradation. The driver-node degradation is defined as the change in the driver-node requirement normalized by the network size:


$$\begin{array}{c} \Delta_{D}=\frac{\left|{𝒟}_{f}|-|𝒟\right|}{n}, \end{array}$$

where 𝒟 and ${𝒟}_{f}$ denote the driver-node sets before and after edge failures, respectively. The matched-node degradation is defined as


$$\begin{array}{c} \Delta_{M}=\frac{N_{M}-N_{M,f}}{\max(N_{M},1)}, \end{array}$$

where $N_{M}$ and $N_{M,f}$ denote the numbers of matched nodes before and after edge failures, respectively. The change in the PDS-size requirement after recomputation is defined as


$$\begin{array}{c} \Delta_{P}=\frac{\left|S_{f}|-|S\right|}{n}, \end{array}$$

where *S* and $S_{f}$ denote the greedy-pruned power dominating sets computed before and after edge failures, respectively. Thus, $\Delta_{P}$ quantifies the change in the number of monitoring nodes required after recomputation and should not be interpreted as a loss of monitoring coverage. For completeness, the change in the number of observed non-PDS nodes associated with the recomputed monitoring configuration is defined as


$$\begin{array}{c} \Delta_{O}=\frac{\left(|O_{before}|-|S|\right)-\left(|O_{after}|-|S_{f}|\right)}{\max\left(|O_{before}|-|S|,1\right)}, \end{array}$$

where *O*_before_ denotes the observed set before edge failures, and *O*_after_ denotes the observed set obtained after recomputing the greedy-pruned PDS $S_{f}$ on the failed graph. This quantity describes the change in the number of observed non-PDS nodes associated with the recomputed monitoring configuration and is not interpreted as an independent measure of monitoring-coverage loss. Actual coverage loss without reconfiguration is evaluated directly by applying the original PDS *S* to the failed network and examining *O*_old_.

In addition, the edge failure ratio is defined as


$$\begin{array}{c} \phi =\frac{\left|E|-|E_{f}\right|}{\left|E\right|}, \end{array}$$

which quantifies the fraction of removed edges and enables direct comparison between structural damage and post-failure changes in the evaluated performance metrics. This formulation is applied to the main normalized performance metrics, namely the driver-node ratio ($r_{D}$), matched-node ratio ($r_{M}$), PDS ratio ($r_{P}$), and observed fraction ($r_{O}$), enabling consistent quantification of post-failure changes across all evaluated metrics. For the intact-network case (*q* = 1), the edge-failure ratio is zero (ϕ=0); therefore, the sensitivity coefficients are undefined. This case is retained only as a baseline and is excluded from all summary statistics involving the sensitivity coefficients.

Monte Carlo simulations with *MC* = 200 independent edge-failure realizations are used to estimate the expected monitoring coverage of the original power dominating set and the expected post-failure driver-node ratio for each network configuration. The summary statistics reported in the tables (mean, median, minimum, and maximum) are computed across all evaluated parameter configurations, reflecting the range of behaviors observed over the considered network sizes, edge densities, and edge-survival probabilities.

### 4.2 Robustness 1uantification

In addition to the primary performance metrics, several derived measures are introduced to systematically quantify and compare the robustness of structural controllability and power-domination-based monitoring under edge failures.

**Failure Sensitivity Coefficient:** The sensitivity of a performance metric to edge removals is quantified using the ratio-based coefficient


$$\begin{array}{c} \kappa_{X}=\frac{\Delta_{X}}{\phi },\;\phi >0, \end{array}$$

where X∈{D,M,P,O} identifies the corresponding performance metric; $\Delta_{X}$ denotes the corresponding post-failure change, and ϕ is the edge-failure ratio. This coefficient provides a normalized indication of the magnitude of each metric’s change relative to the extent of structural damage. Larger values of $\left|\kappa_{X}\right|$ indicate greater sensitivity to edge failures. Cases where ϕ=0 are excluded from this analysis.

**Control–Monitoring Balance:** The trade-off between structural controllability and power-domination-based monitoring after edge failures is captured by


$$\begin{array}{c} \Gamma =\frac{r_{D,f}}{r_{P,f}},\;r_{P,f}>0, \end{array}$$

where $r_{D,f}=N_{D,f}/n$ is the post-failure driver-node ratio, and $r_{P,f}=|S_{f}|/n$ is the post-failure power domination set (PDS) ratio. Values of Γ≈1 indicate a balanced relationship in which structural controllability and power-domination-based monitoring require similar numbers of nodes. Γ≫1 indicates that structural controllability requires more driver nodes than power-domination-based monitoring requires monitoring nodes, while Γ≪1 indicates the opposite. The ratio is well-defined for all evaluated configurations because $r_{P,f}>0$ in every network realization.

**Control–Monitoring Requirement Gap:** The difference between the post-failure changes in driver-node and monitoring-node requirements is defined as


$$\begin{array}{c} \Lambda =\Delta_{D}-\Delta_{P}, \end{array}$$

where $\Delta_{D}$ and $\Delta_{P}$ quantify the normalized changes in the required numbers of driver nodes and monitoring nodes, respectively. A positive Λ indicates a larger increase in the driver-node requirement than in the monitoring-node requirement, whereas Λ<0 indicates the opposite.

## 5 Results and analysis

This section analyzes the empirical behavior of structural controllability and power-domination-based monitoring under random edge failures across different network sizes and densities. To provide a visual interpretation of controllability structure, Figs 4 and 5 illustrate a representative sparse network realization before and after edge failures, where significant degradation is observed. This example highlights the vulnerability of structural controllability in sparse networks, where edge removals severely disrupt the matching structure, leading to a substantial increase in the number of required driver nodes.

**Fig 4:**
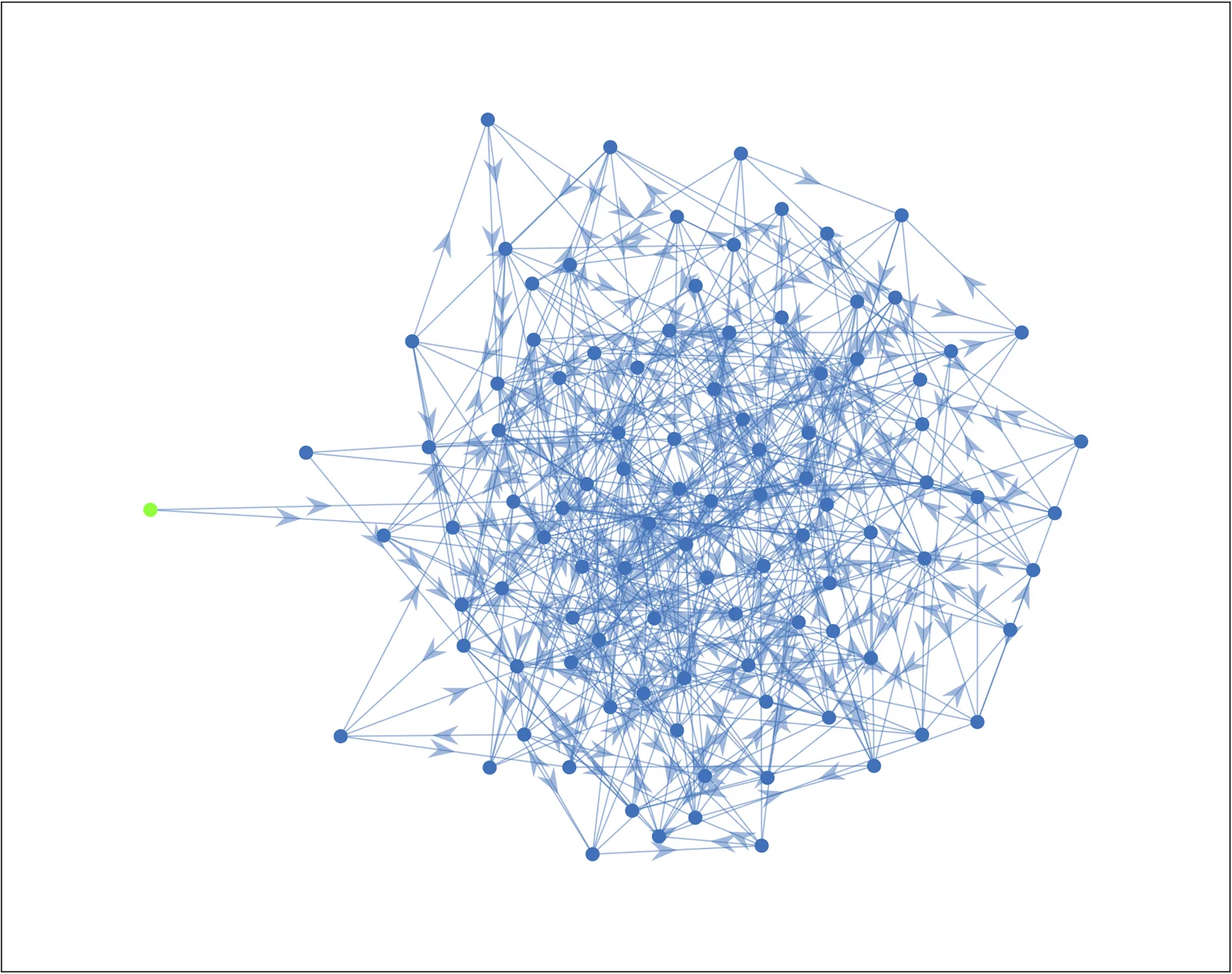
Structural controllability before edge failures in a sparse network. The network requires only one driver node (#*Drivers* = 1), with 99/100 nodes matched, indicating efficient structural controllability through maximum matching.

**Fig 5:**
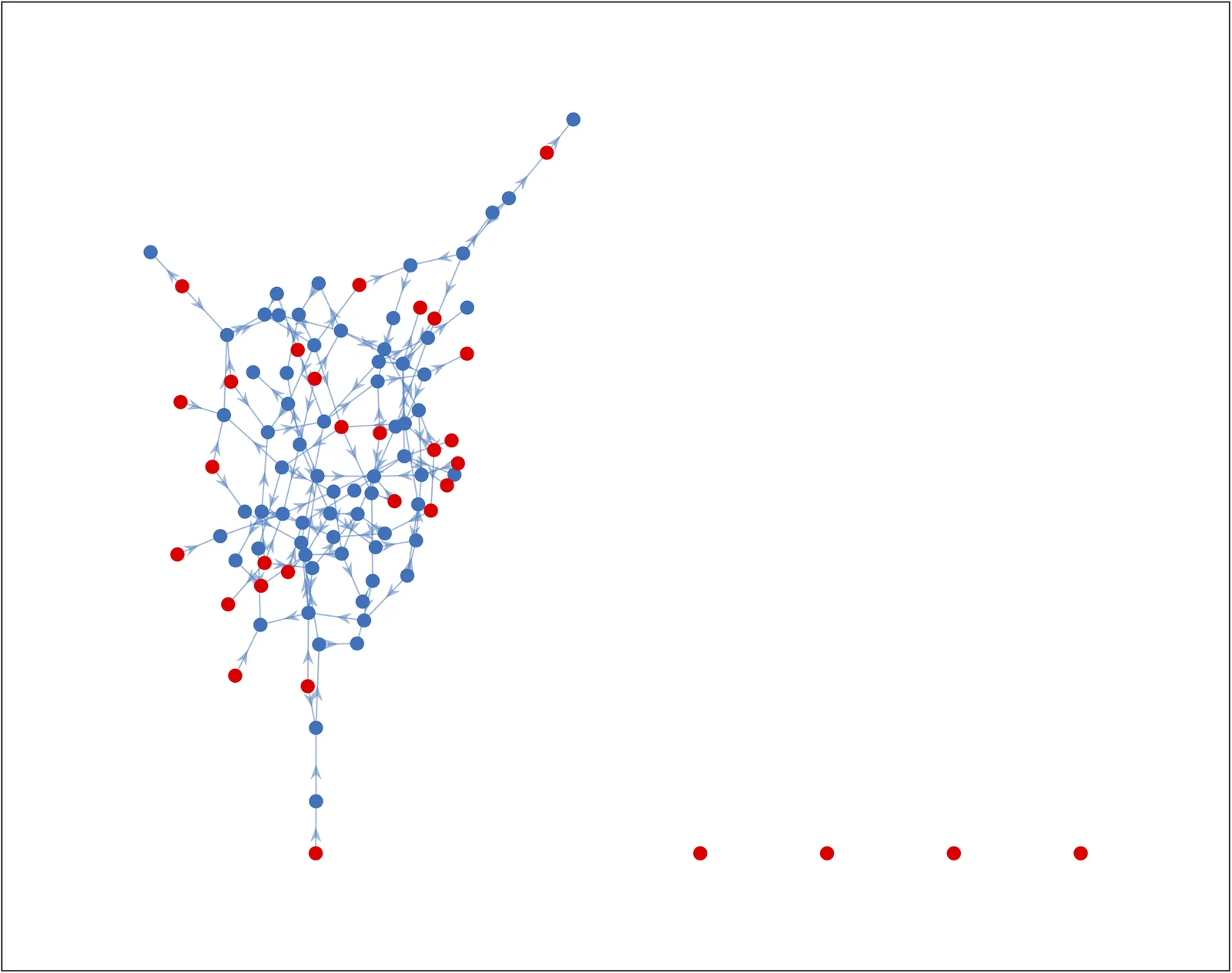
Structural controllability after random edge failures for the same network configuration. Red nodes denote unmatched nodes (driver nodes). The number of required driver nodes increases substantially (#*Drivers* = 31), while only 69 out of 100 nodes remain matched, indicating substantial degradation of the maximum matching structure.

At the same time, it is important to note that such degradation is not uniform across all network densities. For instance, in moderately dense networks, structural controllability remains relatively stable. For certain moderately dense configurations (e.g., *p* = 0.3, *q* = 0.5), the number of driver nodes remains unchanged, with #*Drivers* = 1 both before and after edge failures, reflecting the presence of structural redundancy. This contrast indicates that the robustness of structural controllability is strongly dependent on network density, a behavior that is further analyzed in the Discussion section.

Fig 6 compares the post-failure driver-node and PDS ratios. In sparse network configurations (low edge probability *p*), particularly for small network sizes and severe edge-failure conditions, the driver-node ratio is generally higher than the PDS ratio, indicating a greater post-failure node requirement for structural controllability than for power-domination-based monitoring. As network density increases, or as network size increases under the adopted Erdős–Rényi parameterization with the associated increase in expected degree, both ratios generally decrease and the difference between them becomes smaller, indicating reduced disparity in the node requirements of the two frameworks.

**Fig 6:**
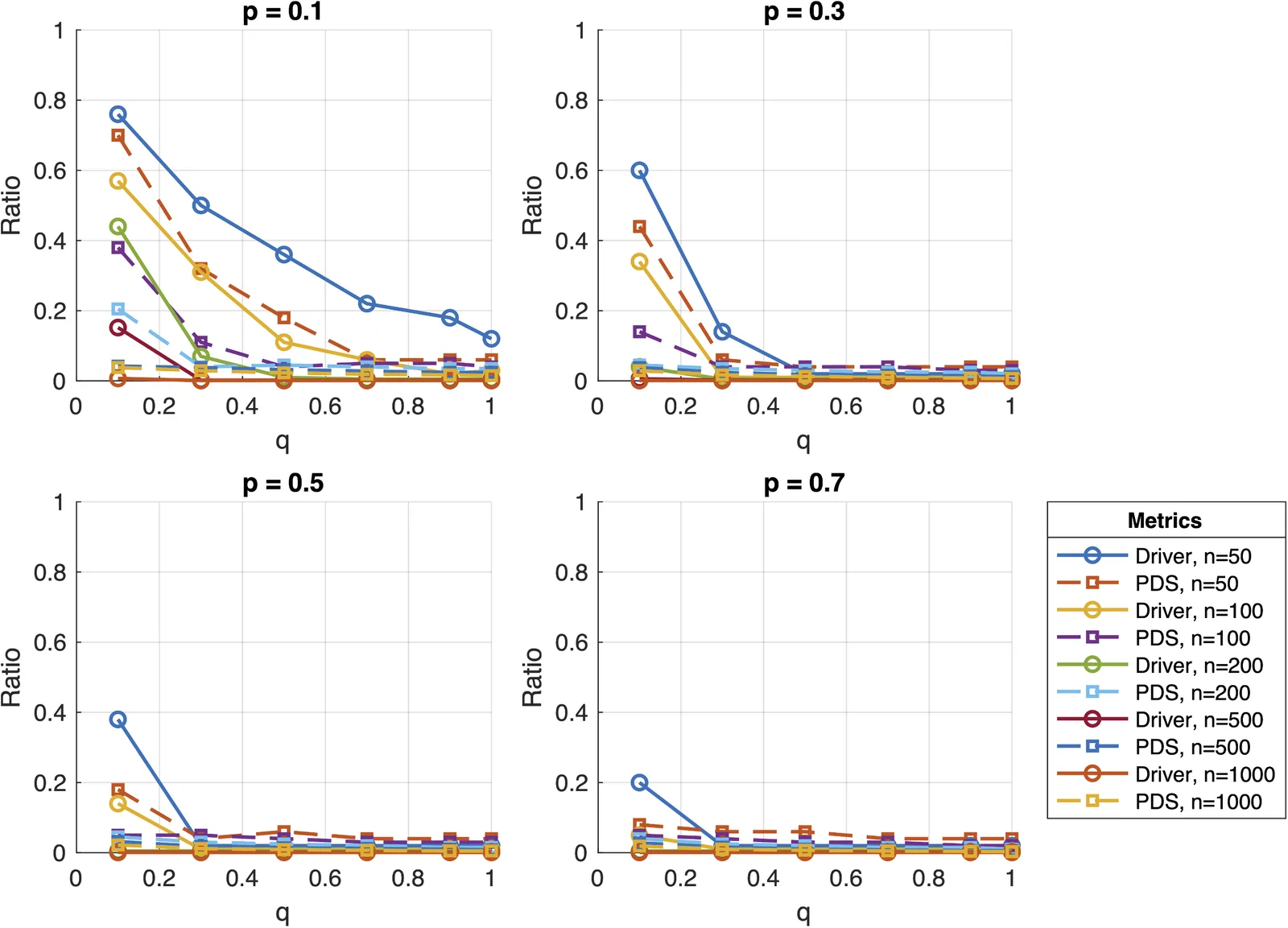
Comparison between the driver-node ratio and power domination set (PDS) ratio after edge failures across different network sizes and densities.

The baseline comparison before failures is presented in Fig 7. In most network configurations, the PDS size exceeds the number of driver nodes, indicating that power-domination-based monitoring generally requires more monitoring nodes than structural controllability requires driver nodes. This relationship is further reflected after edge failures through the control–monitoring balance ratio. The median value (Γ=0.250), which is well below unity, indicates that the PDS size generally remains larger than the corresponding driver-node requirement across the evaluated degraded configurations. Nevertheless, the maximum observed value (Γ=3.667) demonstrates that certain configurations exhibit substantially higher driver-node requirements than monitoring-node requirements, highlighting conditions under which structural controllability is more strongly affected relative to power-domination-based monitoring.

**Fig 7:**
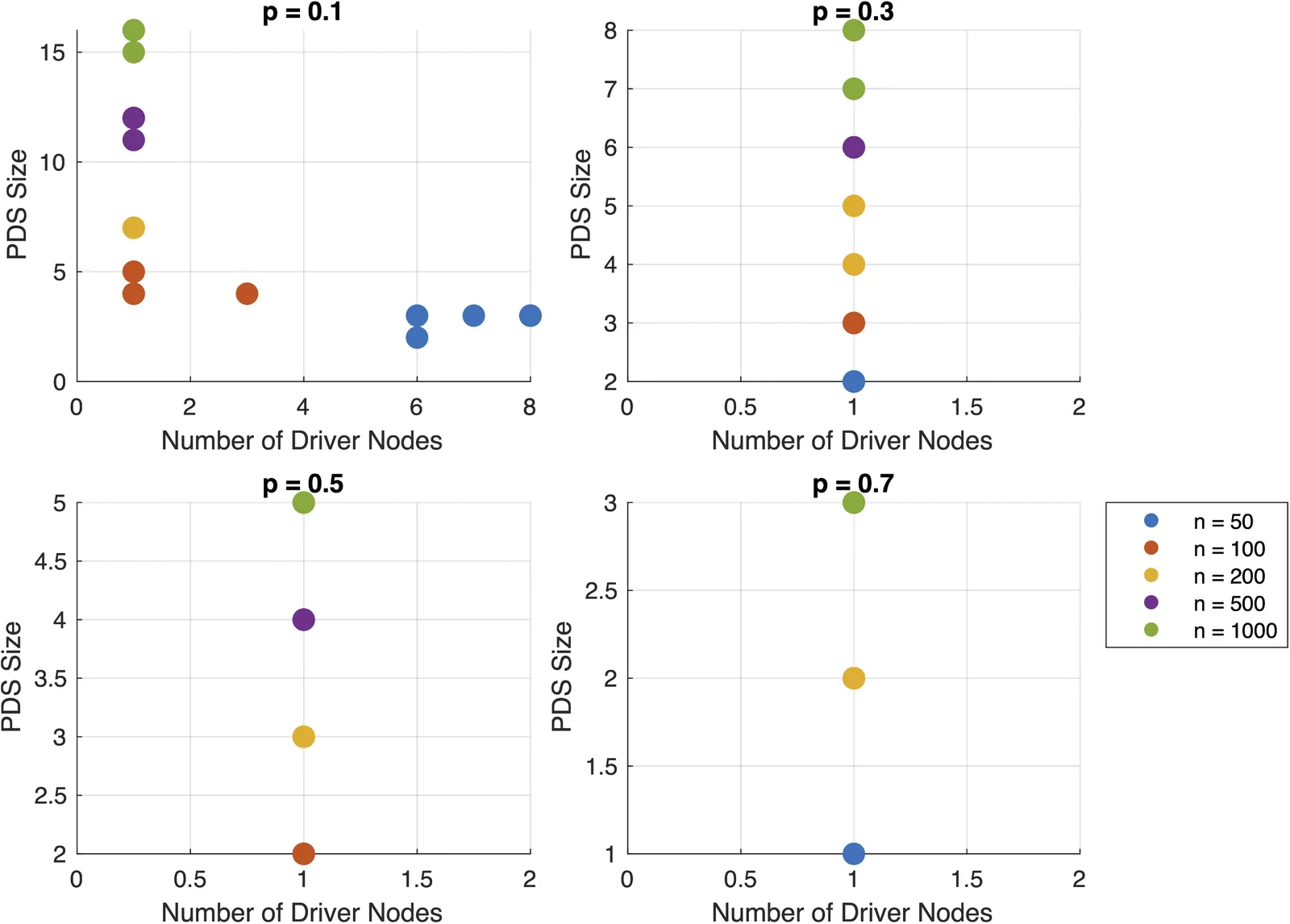
Comparison between the number of driver nodes and PDS size before edge failures for different network sizes and edge probabilities.

The degradation behavior is illustrated in Fig 8. The increase in the driver-node requirement is generally larger than the change in the PDS-size requirement under severe edge failures, particularly in low-density networks, indicating a more pronounced post-failure change in the structural controllability requirement under such conditions. As network density increases, the difference between the two requirement changes generally decreases.

**Fig 8:**
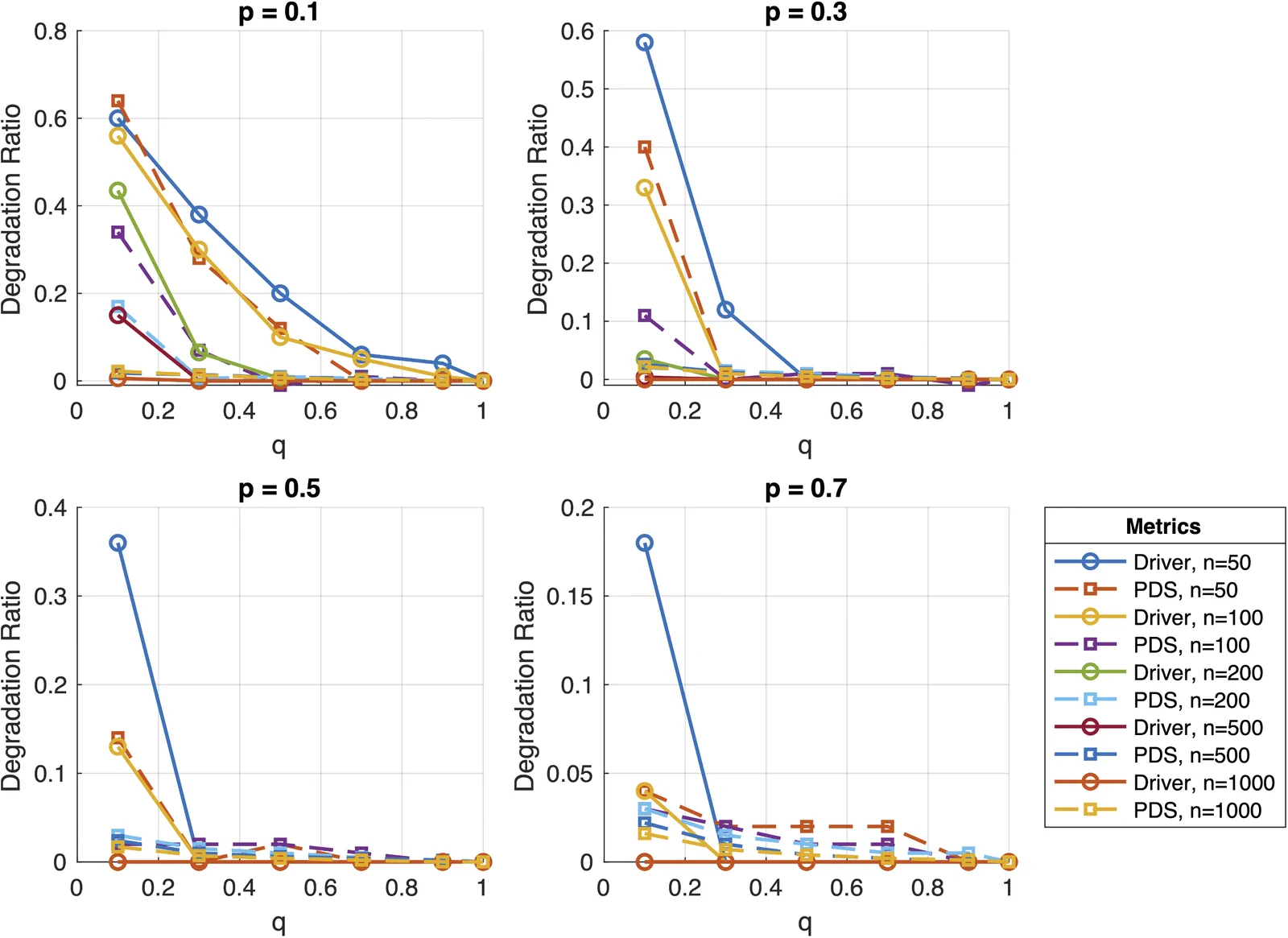
Comparison of degradation ratios between driver-node and PDS metrics under varying edge-survival probabilities.

Fig 9 compares matching degradation with the change in observed non-PDS nodes associated with the recomputed monitoring configuration across different network configurations. The results show that matching degradation is generally more pronounced than the corresponding change in observed non-PDS nodes, particularly in sparse and highly degraded networks. This behavior is consistent with the structural controllability framework of Liu *et al.* [5], where controllability is fundamentally governed by the maximum matching structure of the network. In contrast, the observed non-PDS node metric associated with the recomputed monitoring configuration generally exhibits smaller changes under the same edge-failure conditions.

**Fig 9:**
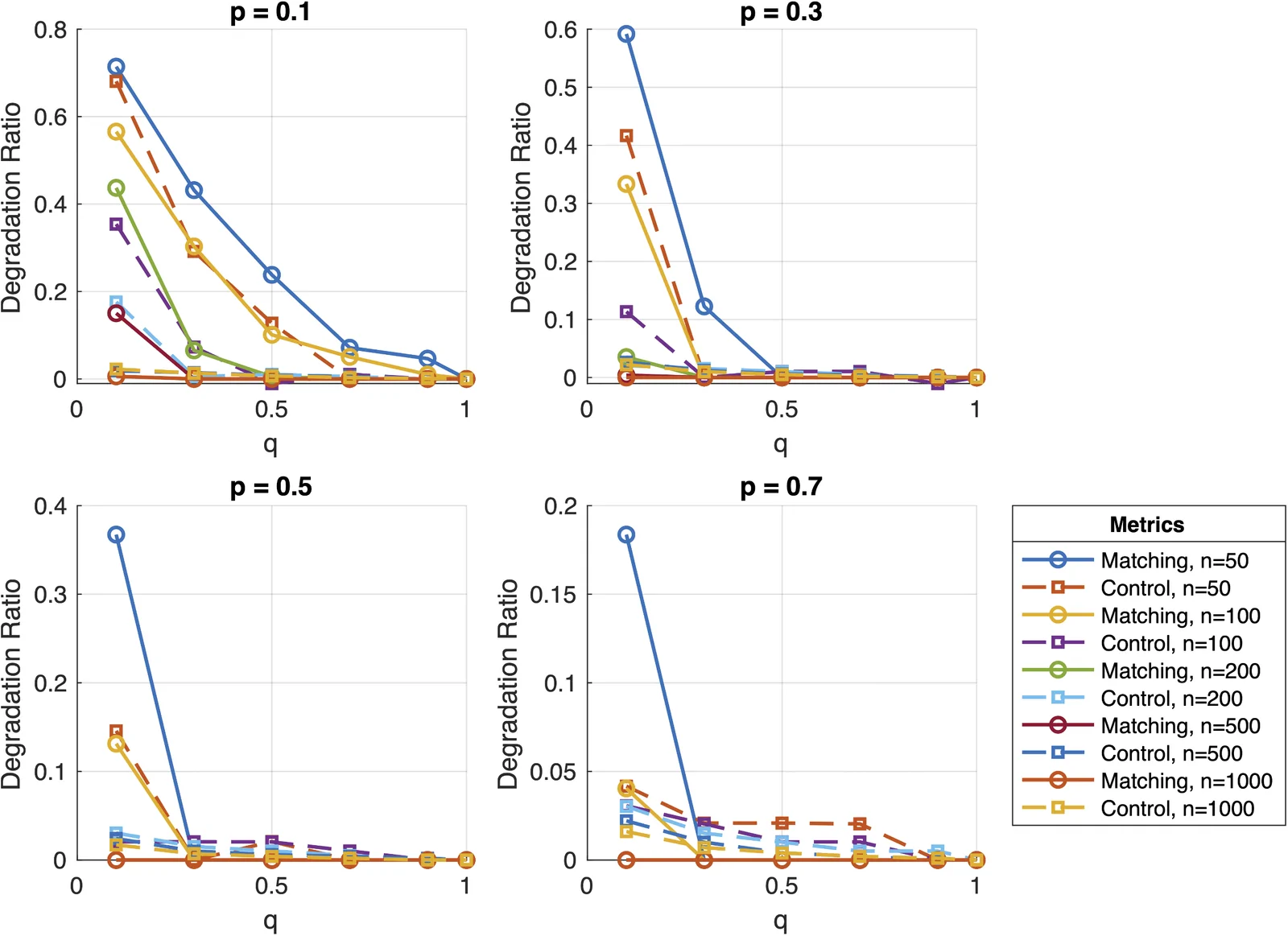
Comparison between matching degradation and the change in observed non-PDS nodes associated with the recomputed monitoring configuration across different network sizes and edge probabilities.

The impact of edge failures is illustrated in Fig 10, where increasing edge failure ratios consistently lead to higher degradation in both structural controllability and power-domination-based monitoring. Notably, dense networks exhibit a near “all-or-nothing” behavior, characterized by stable performance over a broad range of edge-failure conditions, followed by a more pronounced increase in degradation under severe failures. This phenomenon reflects the high structural redundancy of dense networks, where multiple alternative paths help maintain structural controllability and power-domination-based monitoring under moderate structural disruption. This transition is particularly noticeable in high-density configurations (e.g., *p* = 0.7), where degradation remains limited under moderate edge failures but increases more noticeably under severe structural degradation.

**Fig 10:**
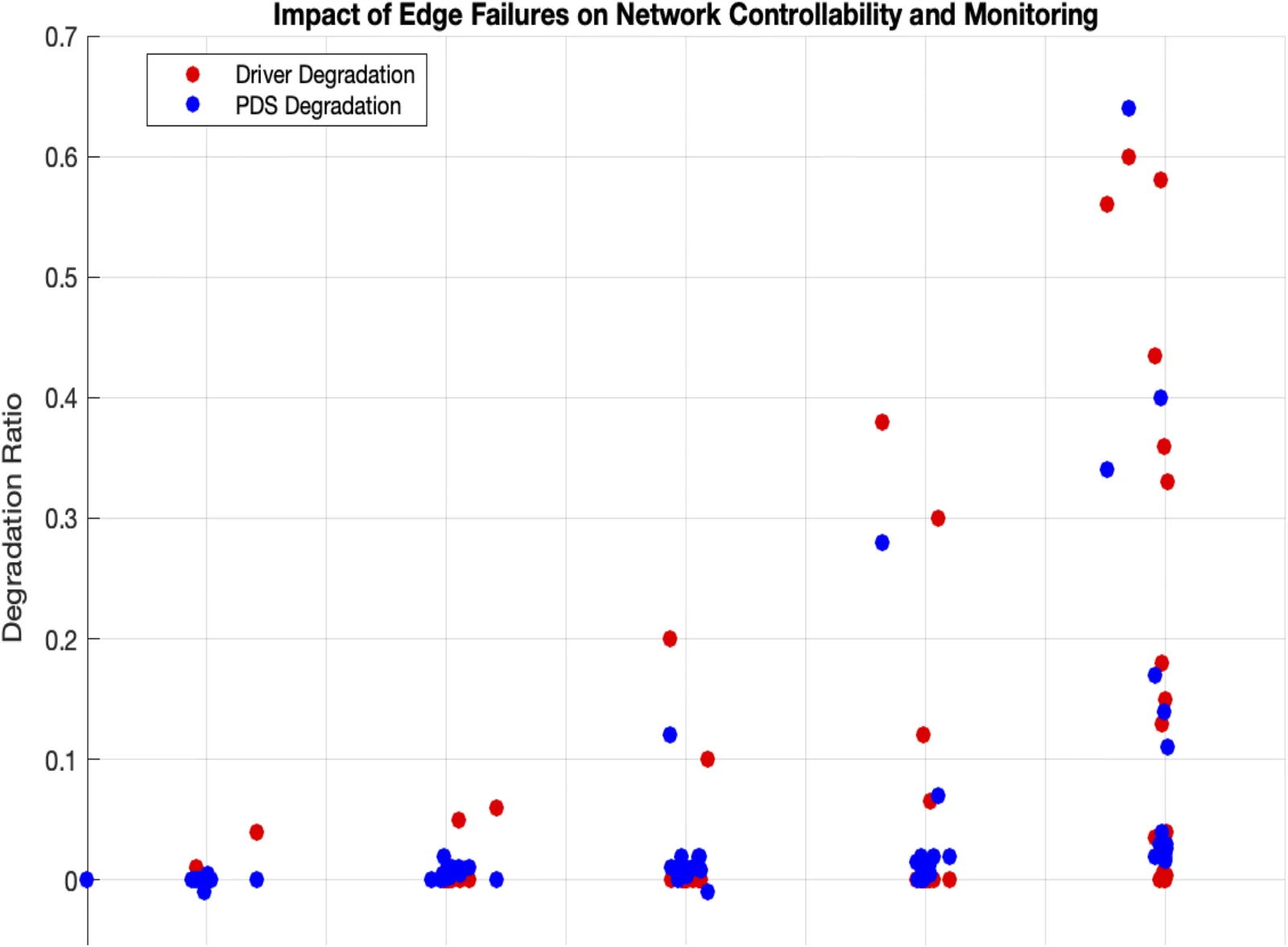
Relationship between edge failure ratio and degradation in structural controllability and power-domination-based monitoring across different network configurations.

The influence of network size is analyzed in Figs 11 and 12. Under the adopted Erdős–Rényi parameterization, increasing network size while holding the edge probability fixed also increases the expected degree; therefore, the observed degradation patterns reflect the combined influence of network size and connectivity rather than a pure network-size effect. In sparse configurations (*p* = 0.1), both the driver-node increase and the PDS-size requirement change generally decrease with increasing network size, whereas higher-density configurations (p≥0.5) exhibit consistently low and relatively stable degradation across the considered network sizes.

**Fig 11:**
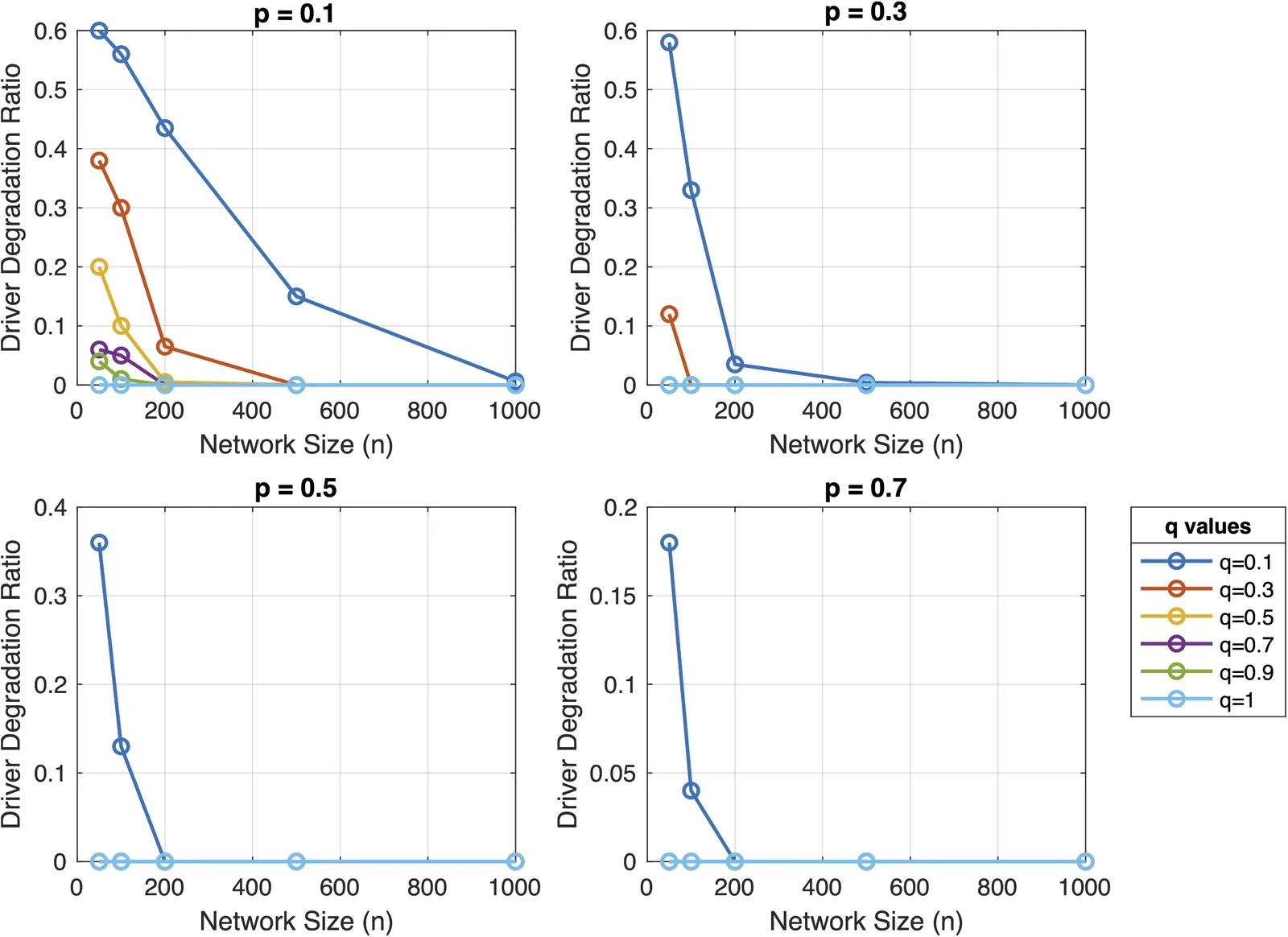
Driver-node degradation ratio across network sizes for different edge probabilities (*p*) and edge survival probabilities (*q*) under the adopted Erdős–Rényi parameterization.

**Fig 12:**
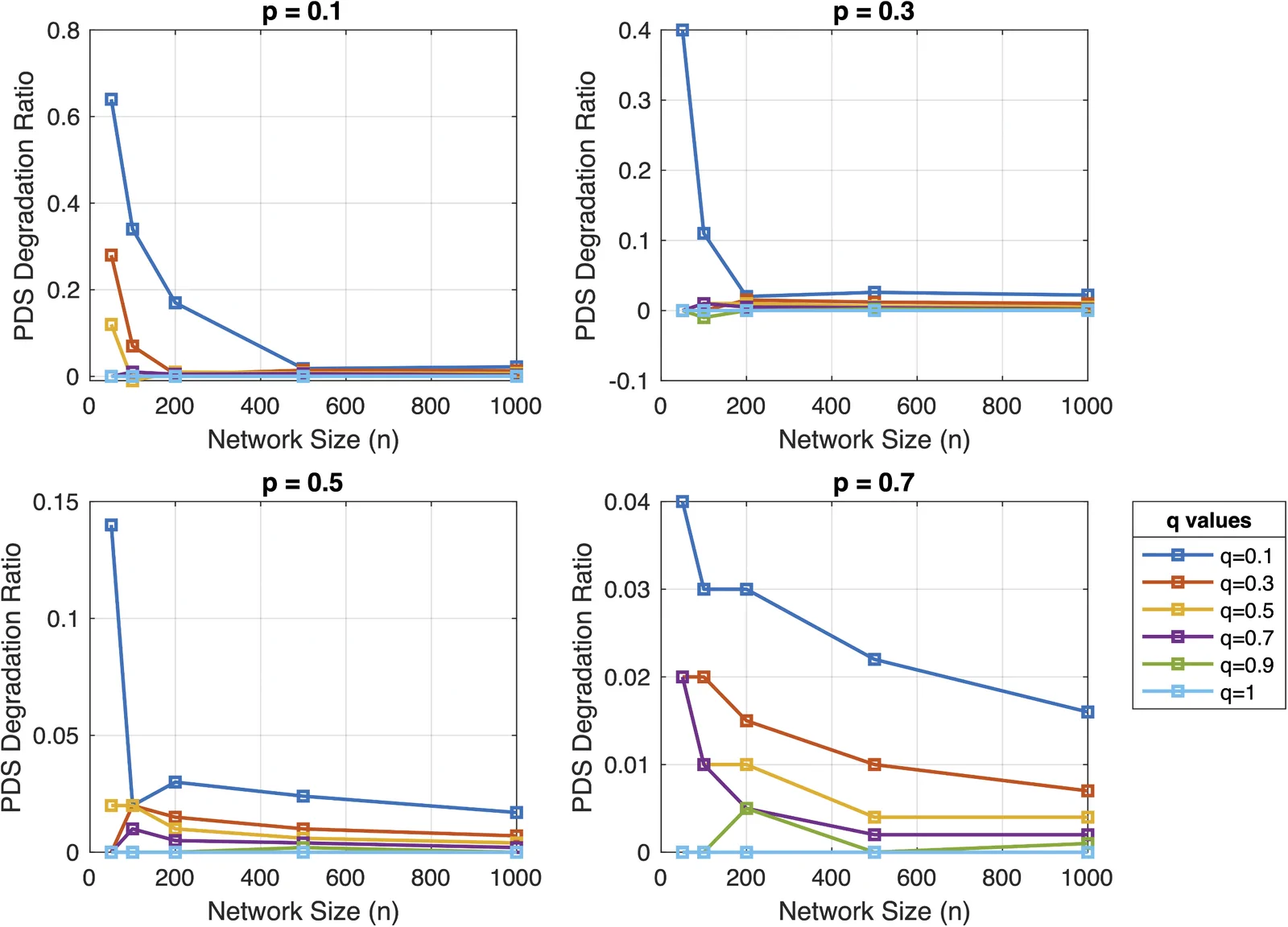
PDS-size requirement change across network sizes for different edge probabilities (*p*) and edge survival probabilities (*q*) under the adopted Erdős–Rényi parameterization.

Fig 13 illustrates the relationship between the post-failure driver-to-PDS ratio and the control–monitoring requirement gap, $\Lambda =\Delta_{D}-\Delta_{P}$, across all network configurations. While most configurations cluster near zero, indicating similar post-failure changes in the driver-node and monitoring-node requirements, the positive requirement gaps generally attain larger magnitudes than the negative ones, whereas negative gaps remain comparatively limited. This asymmetry suggests that, when the increase in the driver-node requirement exceeds the change in the monitoring-node requirement (Λ>0), the disparity between the two measures can become considerably more pronounced, reflecting greater vulnerability of structural controllability relative to power-domination-based monitoring. Overall, these observations are consistent with the greater vulnerability of structural controllability to random edge failures observed throughout the study.

**Fig 13:**
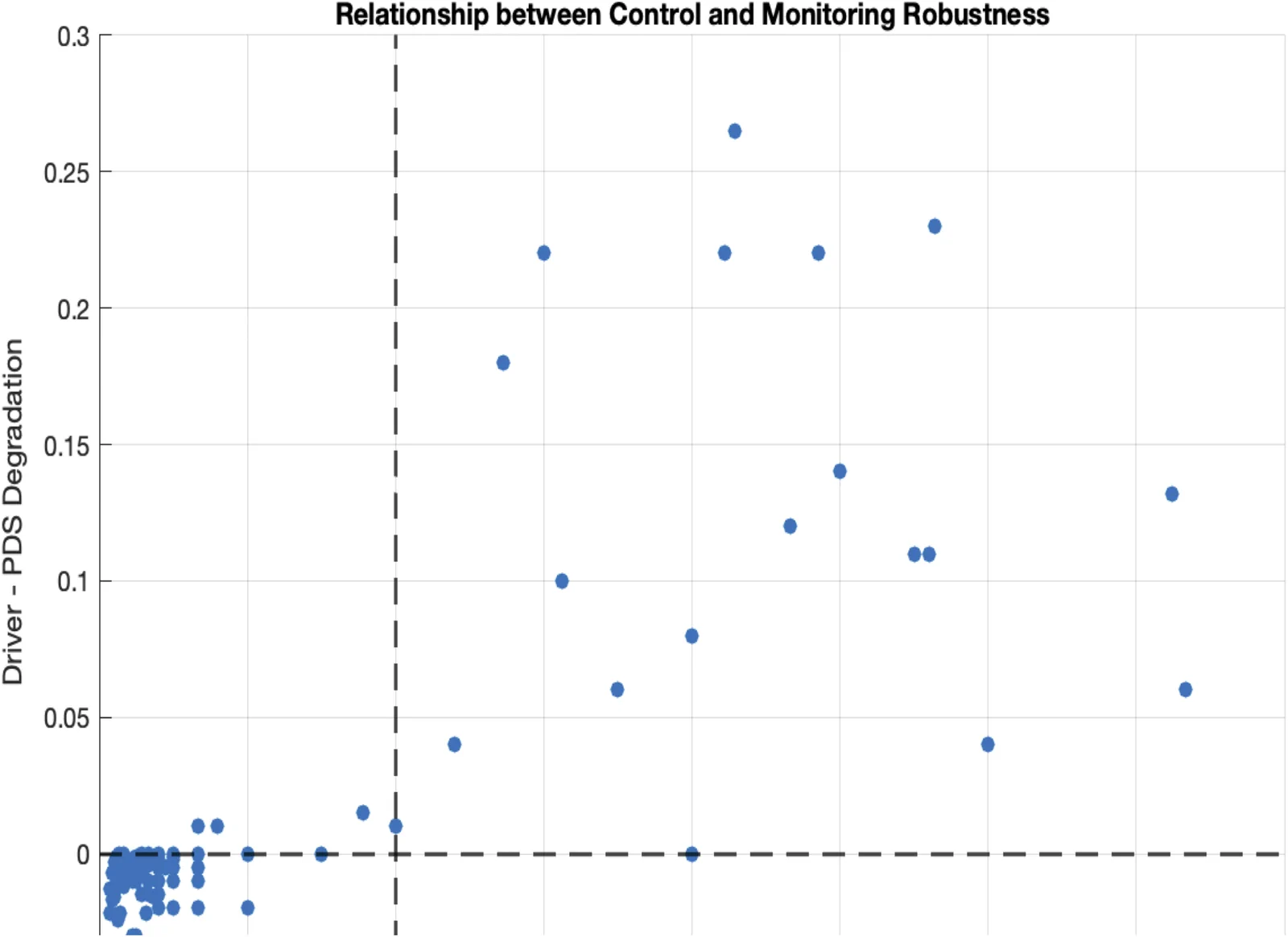
Relationship between the post-failure driver-to-PDS ratio and the control–monitoring requirement gap $\left(\Lambda =\Delta_{D}-\Delta_{P}\right)$ across network configurations. Positive values of Λ indicate a larger increase in the driver-node requirement than in the monitoring-node requirement, whereas negative values indicate the opposite.

To further support these observations, a quantitative analysis based on the derived robustness metrics is conducted. Table 2 summarizes the key robustness metrics across all evaluated configurations, providing a quantitative characterization of the comparative robustness behavior of structural controllability and power-domination-based monitoring. These descriptive statistics complement the trends observed in Figs 8, 10, and 13 by summarizing the overall distribution of the robustness measures.

**Table 2:** Summary statistics of the key robustness metrics computed from the simulation data.

Metric	Mean	Median	Min	Max
$\kappa_{D}$	0.065	0.000	0.000	0.690
$\kappa_{P}$	0.041	0.017	−0.102	0.736
Λ	0.014	−0.002	−0.040	0.265
Γ	0.566	0.250	0.035	3.667

The driver-based sensitivity coefficient ($\kappa_{D}$) has a mean value of 0.065 and a median of 0.000, with values ranging from 0.000 to 0.690. The corresponding PDS-based sensitivity coefficient ($\kappa_{P}$) has a mean value of 0.041 and a median of 0.017, with values ranging from −0.102 to 0.736. Overall, the larger mean value of $\kappa_{D}$ indicates greater degradation sensitivity of structural controllability on average. The corresponding median values and overlapping ranges indicate that the magnitude of this difference varies across network configurations. Nevertheless, consistent with Figs 8 and 10, the most pronounced degradation differences occur in sparse and severely degraded networks, where structural controllability exhibits the greatest vulnerability. Negative values of $\kappa_{P}$ indicate configurations in which recomputing the PDS reduces the required monitoring-set size after structural degradation. The control–monitoring requirement-gap metric $\Lambda =\Delta_{D}-\Delta_{P}$ remains close to zero overall, with a mean of 0.014 and a median of −0.002. The observed range extends from −0.040 to 0.265. The near-zero median indicates that the typical difference between the post-failure changes in the driver-node and monitoring-node requirements is small, whereas the wider positive range indicates that, in specific configurations, the increase in the driver-node requirement can considerably exceed the corresponding increase in the PDS size. This interpretation is consistent with the degradation patterns observed in Figs 8, 10, and 13, particularly under sparse and severely degraded network conditions.

A key insight from these results is that power-domination-based monitoring exhibits reconfigurability under structural degradation. While recomputing the PDS adapts the monitoring configuration to the degraded network, structural controllability is likewise reevaluated by recomputing the maximum matching, which determines the updated minimum driver-node requirement. Under severe structural degradation, this requirement may increase, particularly in sparse networks.

### 5.1 Robustness to random edge orientation

To assess whether the structural controllability results depend on the random orientation assigned to undirected edges, an additional orientation sensitivity analysis was conducted. For each fixed undirected Erdős–Rényi graph and identical edge-failure realization, the edge orientations were regenerated independently 20 times while preserving the same underlying topology and failure pattern. The analysis covered five network sizes, four edge-probability settings, and six edge-survival probabilities. For each (*n*,*p*) configuration, five independent undirected graph instances were generated, giving 5×4×5=100 base graphs. Each graph was then evaluated under all six edge-survival probabilities using one edge-failure realization per (*n*,*p*,*q*) graph case, resulting in 100×6=600 graph/failure cases. For each such case, 20 independent edge orientations were evaluated, yielding 600×20=12,000 orientation realizations. As summarized in Table 3, nearly three-quarters (74.3%) of the analyzed graph/failure cases produced identical degradation values across all 20 random edge orientations (zero standard deviation). Furthermore, the average standard deviation of the degradation metric was only 0.0043, while the maximum observed value was 0.0451, occurring only in the smallest and sparsest network configurations. These findings indicate that random edge orientation introduces only minimal orientation-dependent variability and has a limited influence on the observed degradation trends. Consequently, the main conclusion regarding the robustness asymmetry between structural controllability and power-domination-based monitoring is not attributable to the particular random orientation adopted in the simulations.

**Table 3:** Summary of the robustness analysis with respect to random edge orientation.

Metric	Value
Base graph instances	100
Graph/failure cases analyzed	600
Random orientations per graph/failure case	20
Total orientation realizations	12,000
Graph/failure cases with zero standard deviation	74.3%
Mean standard deviation of degradation	0.0043
Median standard deviation of degradation	0.0000
Maximum standard deviation of degradation	0.0451

## 6 Discussion

The results reveal a clear robustness asymmetry between structural controllability and power-domination-based monitoring under random edge failures. While both processes degrade as structural damage increases, the robustness asymmetry is configuration dependent. Structural controllability exhibits its most pronounced relative vulnerability under sparse and severely degraded network conditions. This interpretation is supported by the higher mean driver-based sensitivity coefficient ($\kappa_{D}=0.065$) relative to the corresponding PDS-based coefficient ($\kappa_{P}=0.041$), together with the degradation patterns observed in Figs 8, 10, and 13. Consistent with these trends, the corresponding median values indicate that the typical disparity across the pooled configurations is limited, whereas the stronger relative vulnerability of structural controllability is concentrated primarily in sparse and severely degraded configurations. A stratified configuration-level comparison provides additional quantitative characterization of this pattern. The proportion of failure configurations with $\Delta_{D}>\Delta_{P}$ reaches 48% for the sparsest networks (*p* = 0.1) and 50% under the most severe failure condition (*q* = 0.1), while substantially lower proportions are observed under denser or less severely degraded conditions. This stratified pattern is consistent with a more pronounced asymmetry under increased network sparsity and failure severity. The control–monitoring requirement-gap metric Λ remains close to zero overall, while its larger positive values indicate that the increase in the driver-node requirement can considerably exceed the corresponding increase in the PDS size under the most challenging conditions. The control–monitoring balance metric Γ provides complementary evidence for this interpretation. Although its median value (Γ=0.250), which is well below unity, indicates that the typical post-failure configuration requires more monitoring nodes than driver nodes, its maximum value (Γ=3.667) demonstrates that certain sparse and severely degraded configurations require substantially more driver nodes than monitoring nodes. Together, these findings reinforce the conclusion that the stronger relative vulnerability of structural controllability is concentrated primarily in sparse and severely degraded network configurations.

From a structural perspective, this difference can be explained by the underlying mechanisms governing each process. Structural controllability depends on the maximum matching structure of the directed network, rendering it sensitive to edge removals that alter this structure and increase the number of unmatched. This observation is consistent with the foundational framework of Lin [4] and its extension to complex networks by Liu *et al.* [5], as well as subsequent studies emphasizing the vulnerability of controllability to structural changes [6,7,14,15]. More specifically, from a graph-theoretic perspective, edge removals reduce local connectivity and may disrupt the underlying matching structure, potentially increasing the number of unmatched nodes in the recomputed maximum matching and consequently increasing the minimum driver-node requirement. Accordingly, the degradation of structural controllability is closely related to disruption of the maximum matching structure, whereas power-domination-based monitoring benefits from local propagation redundancy that allows monitoring to persist even after partial connectivity loss. Even when edges are removed, alternative propagation paths may still exist, providing an additional form of structural redundancy. This propagation-driven mechanism helps explain the lower average sensitivity represented by $\kappa_{P}$ and aligns with established results in power domination and network monitoring [11,27].

An important insight emerging from this study is the role of reconfigurability in power-domination-based monitoring. While the effectiveness of the original monitoring set degrades under edge failures, recomputing the power domination set provides a reconfigured monitoring set for the degraded network, thereby quantifying the monitoring-resource requirements after failure. In parallel, structural controllability is reevaluated after edge failures by recomputing the maximum matching on the degraded network, which determines the updated minimum driver-node requirement. Once the matching structure is disrupted, the recomputed maximum matching may result in an increased minimum driver-node requirement, particularly under severe structural degradation. This difference provides a structural explanation for the observed asymmetry between the two frameworks.

The impact of network structure further reinforces these findings. Sparse networks exhibit gradual but significant degradation, as the removal of a relatively small number of edges can disrupt critical paths required for both structural controllability and power-domination-based monitoring. In contrast, dense networks display a threshold-like behavior, with performance remaining relatively stable over a broad range of edge-failure conditions before degradation becomes more pronounced under severe edge removals. This behavior is consistent with classical results on network robustness, where dense networks benefit from greater structural redundancy [1–3,16].

As illustrated in Figs 11 and 12, the observed degradation patterns reflect the combined influence of network size and edge density under the adopted Erdős–Rényi parameterization. For sparse configurations (*p* = 0.1), both the driver-node increase and the PDS-size requirement change generally decrease with increasing network size, whereas higher-density configurations (p≥0.5) exhibit consistently low and relatively stable degradation across the considered network sizes. These observations highlight the importance of jointly considering network size and edge density when evaluating network robustness.

Compared to existing work, this study provides a unified framework that directly contrasts structural controllability and power-domination-based monitoring under identical structural conditions. While prior studies have typically examined these processes separately, focusing either on structural controllability robustness [14,25] or on power-domination-based monitoring [11,27], the present analysis reveals fundamental differences in their robustness behavior. The proposed sensitivity coefficients and balance metrics enable a systematic quantitative comparison that extends beyond qualitative observations. These structural differences also provide potential practical implications for resilient network design within the adopted Erdős–Rényi random-network model under independent random edge failures. Within the considered topology-based model, these findings suggest that preserving matching structures or accommodating additional control inputs may be particularly relevant under sparse and severely degraded network conditions. In contrast, the results suggest that reconfiguration of the monitoring set may provide greater flexibility for power-domination-based monitoring through its propagation mechanism. Accordingly, these observations represent model-based implications within the considered simulation setting rather than general recommendations for networked systems.

Our findings extend the discussion initiated by Leitold *et al.* [17] on the importance of detailed system representation. While their work showed that incorporating dynamics can significantly influence the number of required driver and observation nodes, our results reveal a configuration-dependent robustness imbalance between structural controllability and power-domination-based monitoring that remains evident under the simplified, undirected, and static network model considered in this study. In particular, the relative vulnerability of structural controllability is most pronounced under sparse and severely degraded network conditions, reflecting the distinct structural mechanisms underlying maximum matching and propagation-based domination. Furthermore, the observed dependence on network density indicates that sparse networks are particularly vulnerable within the adopted Erdős–Rényi random-network model under independent random edge failures, emphasizing the importance of robustness-aware design strategies for such network configurations.

### 6.1 Limitations

The post-failure change metrics, sensitivity coefficients, and comparative robustness measures are evaluated using a representative failure realization for each parameter configuration. In particular, the main large-scale experiment does not average these comparative metrics across multiple independently generated base graphs for each parameter configuration (*n*,*p*,*q*), which limits the extent to which variability across network realizations can be characterized. Consequently, these quantities characterize the selected realization, may retain realization-specific variability, and should not be interpreted as Monte Carlo expectations across repeated realizations. The Monte Carlo analysis is used separately to estimate the expected non-reconfigured monitoring coverage and the expected post-failure driver-node ratio. Extending the analysis to multiple independent base graphs and failure realizations for the degradation and comparative robustness metrics would provide a more comprehensive characterization of their variability and uncertainty.

The analysis is based on Erdős–Rényi random networks, which do not capture the heterogeneity present in many real-world systems such as scale-free or small-world networks. Additionally, the failure model assumes independent random edge removals, whereas practical systems may experience correlated or targeted failures. The study also relies on structural metrics that do not incorporate edge weights or system dynamics. In addition, structural controllability is evaluated using randomly oriented realizations of the underlying undirected networks. Although the orientation sensitivity analysis indicates limited orientation-dependent variability, extending the analysis to networks with intrinsically directed topologies would provide a more realistic assessment of direction-dependent controllability. These limitations motivate extending the present framework to more heterogeneous, intrinsically directed, and dynamically represented networks under broader classes of structural failures.

## 7 Conclusion

This study presented a systematic comparative analysis of the robustness of structural controllability and power-domination-based monitoring under independent random edge failures in the considered Erdős–Rényi random-network model. By combining sensitivity coefficients, a control–monitoring requirement-gap metric, and control–monitoring balance metrics, the analysis provided a direct and quantitative comparison between the two processes under identical structural conditions, highlighting the importance of jointly analyzing control and monitoring capabilities when assessing network robustness.

The results reveal a configuration-dependent robustness imbalance between structural controllability and power-domination-based monitoring. The most pronounced relative vulnerability of structural controllability is observed under sparse and severely degraded network conditions. Following edge failures, both frameworks are reevaluated on the degraded network through their respective structural mechanisms: the power dominating set is recomputed to determine the updated monitoring-node requirement, whereas the maximum matching is recomputed to determine the updated driver-node requirement for structural controllability. The relative magnitudes of the sensitivity measures and the control–monitoring balance indicate that, while typical post-failure configurations require more monitoring nodes than driver nodes, certain sparse and severely degraded configurations can exhibit substantially increased driver-node requirements. Within the considered topology-based model, these findings indicate that the robustness asymmetry arises from the different structural responses of the two frameworks to edge failures and is most pronounced under sparse and severely degraded network conditions.

Future work may extend the proposed framework to more realistic network topologies, such as scale-free and small-world networks, and to broader failure scenarios involving both edge and node failures. In addition, investigating adaptive controllability strategies that incorporate dynamic reconfiguration or auxiliary control mechanisms could provide further insight into mitigating the vulnerability of structural controllability under structural disruptions.
